# Highly integrable planar-structured printed circularly polarized antenna for emerging wideband internet of things applications in the millimeter-wave band

**DOI:** 10.1038/s41598-024-60678-3

**Published:** 2024-05-02

**Authors:** Ubaid Ullah, Slawomir Koziel, Anna Pietrenko-Dabrowska

**Affiliations:** 1 Networks and Communication Engineering Department, Al Ain University, P.O.Box (112612), Abu Dhabi, UAE; 2 Engineering Optimization and Modeling Center, Reykjavik University, Reykjavik, Iceland; 3 Faculty of Electronics, Telecommunications and Informatics, Gdansk University of Technology, 80-233 Gdańsk, Poland

**Keywords:** Internet of things, IoT devices, Single-layer antenna, Millimeter-wave, IoT application, Wideband designs, Circular polarization, Engineering, Electrical and electronic engineering

## Abstract

This paper proposes a numerically and experimentally validated printed wideband antenna with a planar geometry for Internet of Things (IoT) applications. This design tackles the challenges associated with deploying IoT sensors in remote areas or across extensive geographical regions. The proposed design exploits a coplanar-waveguide-fed modified microstrip line monopole for excitation of circularly polarized waves radiating in the broadside direction. The primary design is based on perturbations of the microstrip line protracted from a grounded coplanar waveguide. The capacitively coupled short rectangular stubs are periodically inserted alternately and excited asymmetrically on each side of the microstrip line parallel to the direction of the electric field vector. The sequential phase excitation of the periodic stubs generates a rectangular-cascaded electric field, which suppresses the stop band at the open end. As a result, the antenna radiates in the broadside direction. The impedance bandwidth of the antenna exceeds 8 GHz in the 28 GHz mm-wave band, i.e., it ranged from 25 to 33.5 GHz. Additionally, an axial ratio below 3 dB is achieved within the operating band from 26 to 33.5 GHz with the alterations of the surface current using straightforward topological adjustments of the physical parameters. The average in-band realized gain of the antenna is 10 dBic when measured in the broadside direction. These results indicate that the proposed design has the potential to improve the connectivity between IoT devices and the constantly varying orientation of satellites by mitigating the polarization mismatch.

## Introduction

The forthcoming generation of wireless communication is in high demand for wide bandwidth catering to the needs of data rates in the range of gigabytes per second^1^. The mm-wave band has proven its potential to provide a balance between reasonable-sized circuits and wide impedance bandwidth ensuring high data rates^2–4^. Additionally, the scarce spectrum availability in the currently utilized band has paved the way for migration to higher frequencies, which is now utilized for the fifth-generation networks^5^. At the millimeter wave band, the data rates are expected to be very high due to the availability of larger bandwidths and higher spectral efficiency^5,6^. To achieve the full potential of this band, high-gain antennas/antenna arrays are required to maintain signal integrity and information reliability, mainly in IoT applications in remote areas^7^. The exponential growth in the number of devices connected to the internet demands further performance enhancement at the front end of the system.

In this regard, satellite communication could emerge as a crucial enabler for the worldwide expansion of IoT connectivity. To realize this concept, the antennas are required to exhibit sufficiently wide impedance bandwidth with a stable directional beam for maintaining a good connection between many devices^8^. In urban and modern metropolitan areas, the problem of signal attenuation and path loss is aggravated by the multipath effects, which pose a grave challenge, particularly in the mm-wave band. However, this can be mitigated at the system level by deploying circularly polarized (CP) antennas instead of linearly polarized devices^9–11^. CP waves are known to have high signal integrity due to their ability to counter the effects of multipath losses, signal attenuation, absorption losses, and signal interference^12^. Excitation of equal magnitude and orthogonal fields with 90-degree phase differences are required for antennas radiating CP waves. This is a challenging undertaking due to the involvement of a complex geometrical modification of the antenna radiator and/or the feeding network.

There exists a rich literature on the CP antenna designs for IoT applications in the conventional lower frequency spectrum below 6 GHz^13–17^. Nevertheless, for the mm-wave band, the antenna’s electrical characteristics are very sensitive to any geometrical parameter changes due to short wavelengths^18,19^. Therefore, it is highly desirable to reduce the geometrical and henceforth the electrical complexities of the antennas, particularly the feeding mechanism, to expedite the design and production process. To address this issue, geometrically simple designs were introduced by different research teams ^3,12,16,17,20–23^. In^12^, a microstrip line antenna excited by a coaxial feed through a hole from the ground plane is designed. Several other antennas were designed based on microstrip line radiators for different frequency bands. The shortcomings of these designs are their linear polarization, narrow impedance bandwidth, and complex circuitry, therefore, their applicability for IoT devices and applications is limited. Moreover, these designs are generally low profile and geometrically simple but operate in a very narrow impedance bandwidth. On the other hand, wideband antennas based on the microstrip radiator were also proposed in the literature. For instance, in^13^ a linear series-fed antenna array with a relatively broad band of ~ 20% has been reported. However, the structure is geometrically complex and involves additional phase shifters for achieving wideband performance. Another wideband design was proposed in^14^, which is based on the concept of multilayers with reflectors, thereby adding to the circuit complexity as well as production cost. For modern wireless communication systems, high data rates with reliable signal quality are required, which emphasizes the importance of wideband antennas with circular polarization^24–26^. Minimizing the complexity of the circuit while maintaining high performance is also vital for realizing an easy-to-implement design.

This paper presents a wideband, circularly polarized antenna printed on a single substrate. The proposed single-port antenna is excited using a grounded coplanar waveguide-fed microstrip line. The extended microstrip line is supplemented with capacitively coupled periodic stubs systematically placed in pairs on both sides of the line forming a series-fed array antenna. The asymmetrical position and a slight offset in the protruded stubs alter the fringing electromagnetic fields by canceling the out-of-phase current components resulting in radiation of the electromagnetic waves from the antenna. The narrow gap between the stubs and the feedline provides a path for the currents to flow on the opposite edges of the stubs resulting in current addition and cancellation with phase progression resulting in circular polarization. The proposed design is easy to realize due to the simple topology printed on a single substrate. The antenna features a wide impedance bandwidth of more than 8 GHz while maintaining axial ratio below 3 dB from 26 to 33.5 GHz, which corresponds to approximately 7.5 GHz bandwidth. Additionally, a stable radiation pattern in the broadside direction ensures almost a flat gain making the antenna suitable for multiple applications in the mm-wave band. The planar geometry and topological simplicity of the feeding circuit and the radiator enable conformability and easy integration of the antenna. The novelty and the methodological contributions of the work can be briefly summarized as follows: (i) development of a planar-structured, single-series-fed linear array antenna featuring simple geometry; (ii) excitation of orthogonal field required for circular polarization using a geometrically simple method; (iii) demonstrating wide impedance matching and axial ratio bandwidth with stable in-band realized gain in the broadside direction.

### Single-port series array antenna design and analysis

In the following sub-sections, the proposed antenna is first explicated from the design viewpoint. Subsequently, a detailed analysis is presented, followed by an explanation of the radiation mechanism, and excitation of circularly polarized waves. The analysis is performed theoretically and validated with the help of numerically generated surface current distribution at different angular time instances.

#### Antenna design

**Figure 1:**
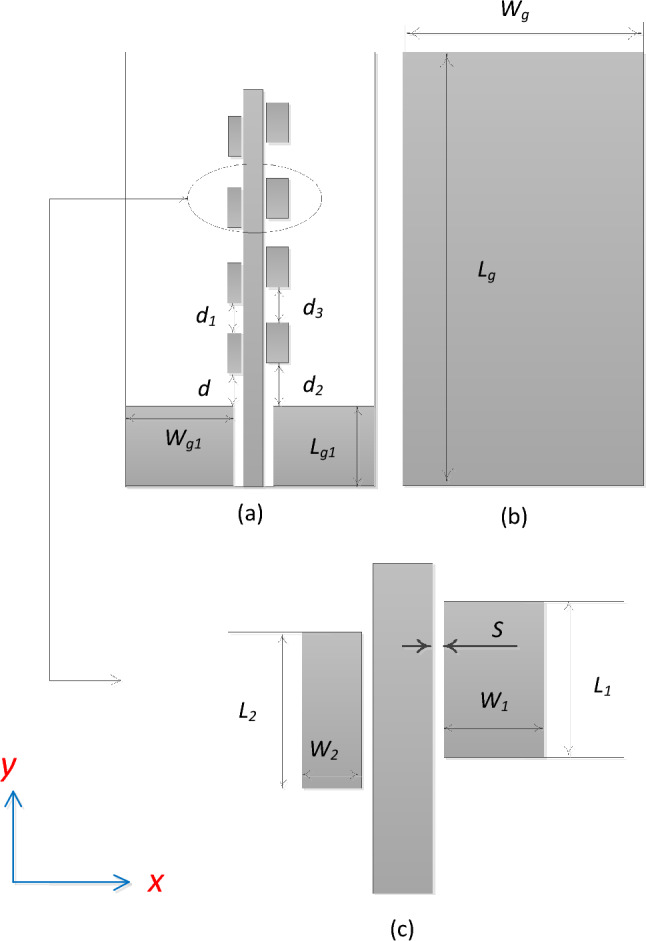
Geometry of the proposed single-layer mm-wave antenna (white: substrate, dark gray: metal): (**a**) parameterized front view, (**b**) back view, (**c**) enlarged view of the capacitively coupled stubs.

The parametrized architecture of the proposed series-fed array antenna has been illustrated in Fig. 1. The antenna is excited using a coplanar waveguide feeding with the full ground plane at the bottom of the substrate. The design is implemented on a single 0.508-mm-thick RO4003C substrate (*ε*_*r*_ = 3.38). Figure 1a depicts the top view of the proposed antenna which is comprised of the two coplanar grounds and an extended microstrip feedline of length (*L*_*m*_) loaded with stubs. The width (*W*_*m*_) of the microstrip line is determined to achieve 50-Ω input impedance. The pair of stubs are placed in close proximity to the microstrip line ensuring strong magnetic coupling between the fields generated by the traveling wave and the stubs. Figure 1b shows the sketch of the rear view and physical dimensions of the fully metalized bottom of the substrate. Moreover, the enlarged pair of stubs is shown in Fig. 1c, which is labeled with the parameters characterizing the physical proportions of the stubs. The geometrical parameters of the antenna are adjusted using multi-stage optimization. In the first stage, the impedance bandwidth of the antenna is optimized followed by the axial ratio optimization. The list of the optimized geometrical parameters (in millimeters) is given in Table 1.

**Table 1:** Optimized parameter values.

Parameter	Value (Millimeters)	Parameter	Value	Parameter	Value (Millimeters)
*W*_*g*1_	5.652	*d*	2.5138	*L*_1_	2.932
*L*_g_	34.476	*d*_1_	1.8610	*W*_1_	1.234
*W*_*g*_	13.360	*d*_2_	3.3179	*L*_2_	3.265
*L*_*m*_	30.163	*d*_3_	2.2796	*W*_2_	0.8293
*W*_*m*_	1.050	*L*_g1_	6.557	*S*	0.012

#### Radiating elements and antenna operation

**Figure 2:**
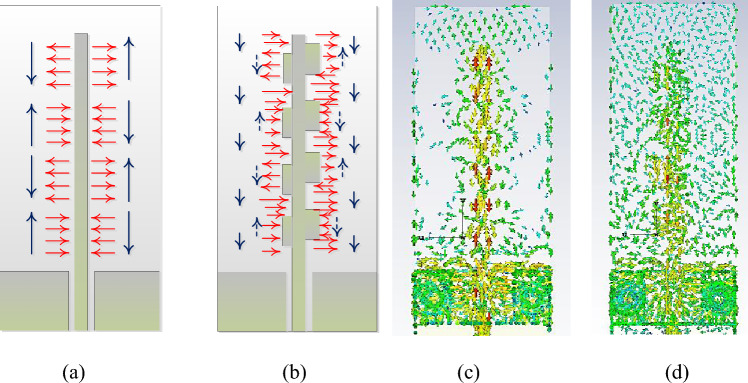
Equivalent fringing field distribution of the microstrip line. Electric field (red), magnetic fields (blue): (**a**) left: normal field distribution, (**b**) right: amplitude tapered field distribution, (**c**) microstrip line surface current, (**d**) surface current with stubs.

To explain the radiation mechanism of the proposed antenna, it is imperative to recall the transmission line theory. In the grounded coplanar waveguide, the electromagnetic fields of the wave propagate along the length of the microstrip line. Contrary to the microstrip line feeding, the coplanar waveguide with the full ground plane at the bottom of the substrate concentrates the field between the microstrip extended from the coplanar grounds and the bottom ground plane. These symmetric electric (E) and magnetic (H) fields are periodic with equal magnitudes but opposite directions resulting in field cancellation along the length of the microstrip line as illustrated in Fig. 2a. The simulated surface current on the feedline is shown in Fig. 2c which clearly illustrates the 180-degree out of phase periodic fields. In this work, through a systematic topological alteration of the microstrip line, amplitude tapering is implemented that breaks the symmetry of the electromagnetic fields. The sketch of the equivalent E and H fields is shown in Fig. 2b. The capacitively coupled pair of stubs are positioned on the field maxima at every half (guided) wavelength at slightly different frequencies in the 28 GHz band. The solid blue arrows show the equivalent in-phase vector magnetic field, whereas the dashed arrows show the fields that are out-of-phase. The in-phase field components are constructively added resulting in the radiation of the antenna. The different physical sizes of the stubs on each side of the line ensure the wideband operation of the device. The polarization of the antenna is further elaborated in the next section based on the simulated surface currents.

#### Single element design

**Figure 3:**
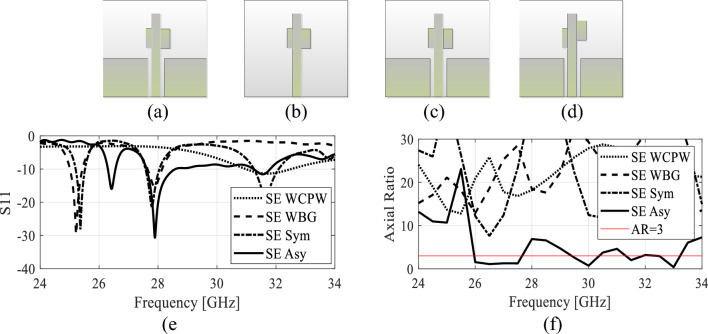
The single element antenna design and its electrical characteristics; (**a**) SSE with CPW only, (**b**) SSE with bottom ground only, (**c**) SSE with both the grounds, (**d**) ASE with both the ground, (**e**) *S*_11_, (**f**) AR.

**Figure 4:**
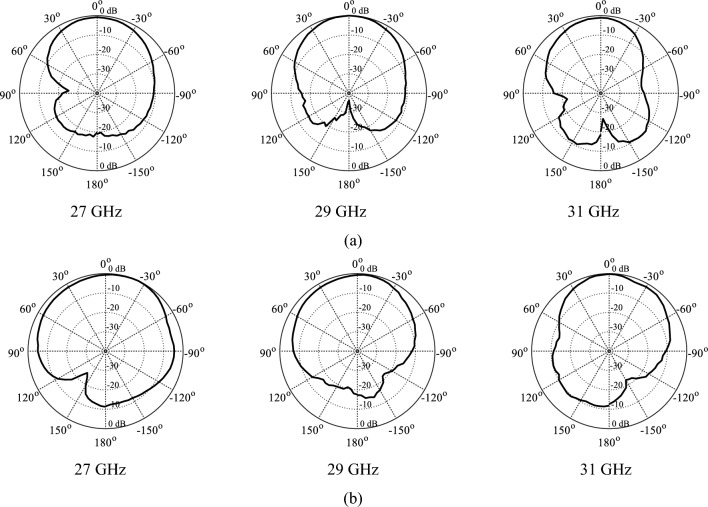
Radiation pattern of the single element design (*xz*-plane) top and (yz-plane) bottom, (**a**) *xz*-plane, (**b**) *yz*-plane.

**Figure 5:**
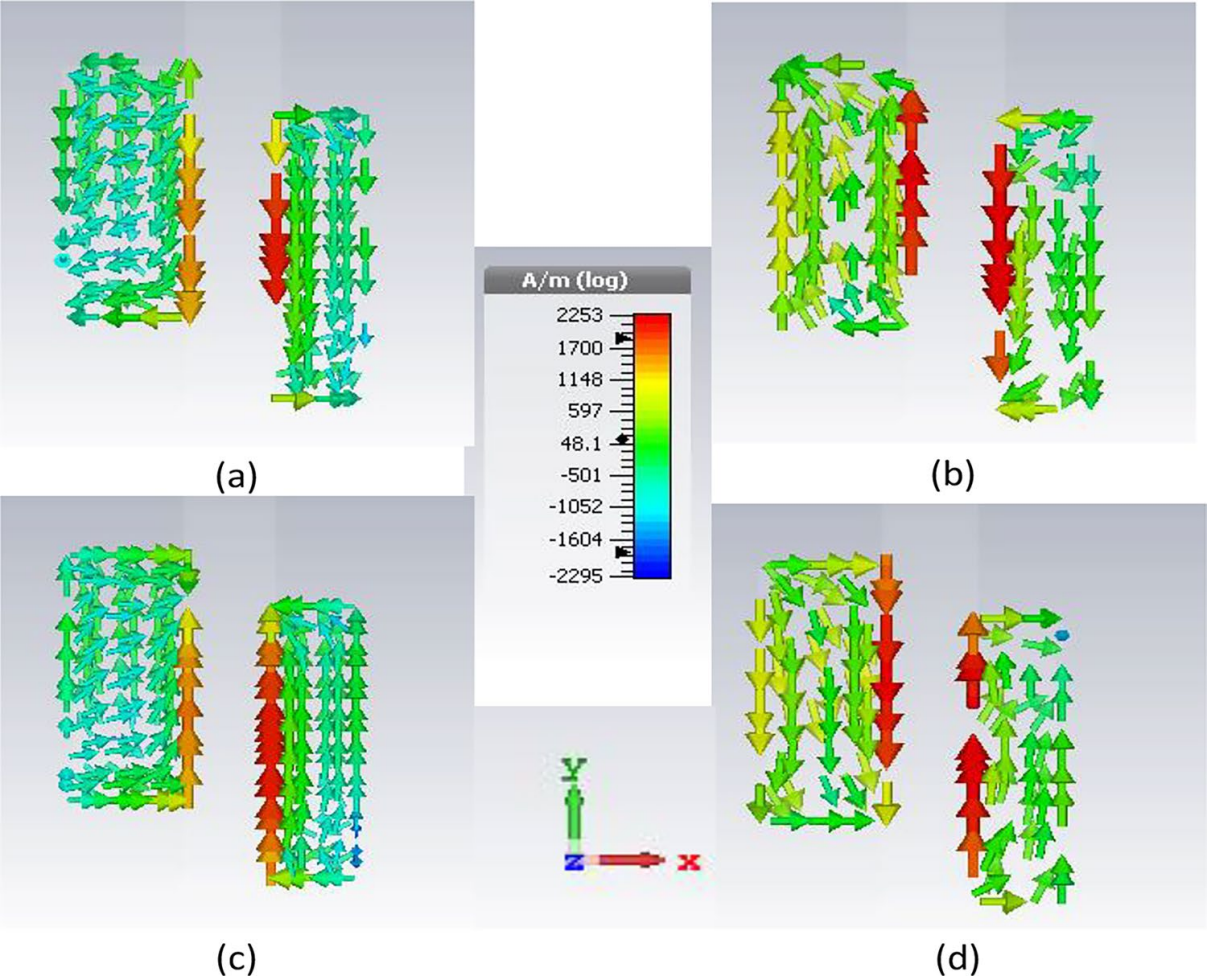
Simulated surface current distribution on the capacitively coupled stubs illustrating left-hand circular polarization: (**a**) 0 degrees, (**b**) 90 degrees, (**c**) 180 degrees, (**d**) 270 degrees.

To understand the antenna operating behavior, the electrical characteristics of a single pair of stubs are first analyzed. As mentioned in the previous section, a pair of stubs is placed on each side of the microstrip line. The length of the stubs (patches) are approximated using the microstrip patch antenna calculator while its width is heuristically selected and later optimized simultaneously. The Four different configuration of the single element are studied which are shown in Fig. 3. In the first case, the symmetric single element (SSE) design with only CPW ground plane is analyzed which is depicted in Fig. 3a. Here, the pair of stubs are symmetrically placed on both sides of the microstrip line. In the second and third case, the electrical characteristics of the SSE with only bottom ground plane (Fig. 3b) and with both the ground planes (Fig. 3c) are analyzed. In the fourth case, the asymmetrical single element (ASE) design with both the ground planes as illustrated in Fig. 3d is analyzed. Furthermore, the *S*_11_ response of the single-element design is depicted in Fig. 5. It is observed that the impedance bandwidth of the single element closely resembles the final antenna design. The reflection coefficient (*S*_11_) and axial ratio of the SSE and ASE antenna are depicted in Fig. 3e and f. The impedance matching is poor when only coplanar ground planes were used with a weak resonance near 31 GHz. It should be noted that AR of the antenna for this case is well above 20 dB. The impedance matching with the bottom ground plane only shows resonance in the 28 GHz region which is attributed to the small size difference between the stubs that causes some constructive correlation of the currents. The AR for this configuration of the antenna also does not meet the requisites as seen in Fig. 3f. Similarly, the SSE with both the ground planes (legend: SE Sym) shows a very narrowband impedance matching and poor AR within the band of the interest. Finally, the ASE with both the ground planes is analyzed in terms of both the performance figures. The *S*_11_ response indicate a wideband response and improved impedance matching and AR compared to the symmetrical configuration of the single element design. The geometrical parameters of the design are later optimized simultaneously. The radiation pattern of the single element is also analyzed in Fig. 4 at multiple frequencies as shown. The radiation pattern of the single element shows a broadside radiation pattern throughout the operating bandwidth of the antenna in both the *xz*- and *yz*-plane which are illustrated in Fig. 4a and b, respectively.

#### Circular polarization

To further illustrate the polarization mechanism of the antenna, the surface current is shown on the capacitively coupled pair of stubs near the CP center frequency at 29 GHz. The orientation of the field with phase progression clearly indicates the rotation of the vector field in the clockwise direction yielding left-hand circularly polarized (LHCP) in the broadside direction. Figure 5a and b indicate the position of the vertical fields at 0 degrees and 180 degrees in the *y*-direction and –*y*-direction, respectively. Similarly, Fig. 5b and d show the fields on the horizontal edges of the stubs in the *x*-direction and −*x*-direction at 90 degrees and 270 degrees. This clearly indicates that the field orientation changes with the change in the angular time, resulting in the excitation of circular polarization. It is important to note that no active circuit elements or polarization converters are used in the proposed design, which makes it a very cost-effective, and geometrically simple antenna with wide impedance and axial ratio bandwidth.

#### Design evolution

**Figure 6:**
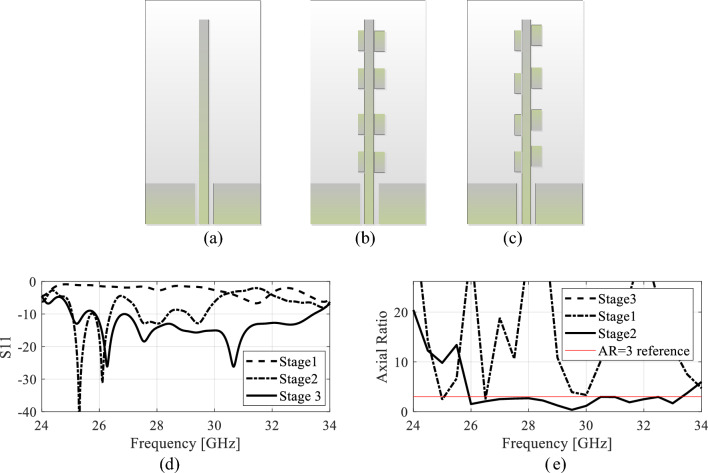
Design evolution stages and the electrical characteristics of the antenna design stages; (**a**) stage 1, (**b**) stage 2, and (**c**) stage 3, (**d**) *S*_11_, (**e**) AR.

The design evolution stages are illustrated in Fig. 6. In the first stage, the CPW-to-microstrip line transition is designed and simulated. The geometry of the first stage is shown in Fig. 6a. The impedance matching of the antenna for this stage is shown in Fig. 6d. The *S*_11_ response indicates that the antenna at this stage is not radiating energy into the free space. Similarly, the AR response shown in Fig. 6e indicates linear polarization. In the second stage of the design, the stubs are placed periodically on both sides of the microstrip line as shown in Fig. 6b. With the introduction of the stubs, the field symmetry is broken resulting in the field constructive correlation. The *S*_11_ response of the second stage shown in Fig. 6d illustrates *S*_11_ below − 10 dB within the operating band of the antenna. It should be noted that the antenna parameters are not optimized at this stage. In the final design stage, a slight offset between the two stubs is introduced and all the geometrical parameters are simultaneously optimized using a full-wave multi-stage optimization. The impedance matching and the AR of the final design stage after optimization are also shown in Fig. 6d and e. Both the impedance bandwidth and AR bandwidth reflects wideband response of the antenna.

**Figure 7:**
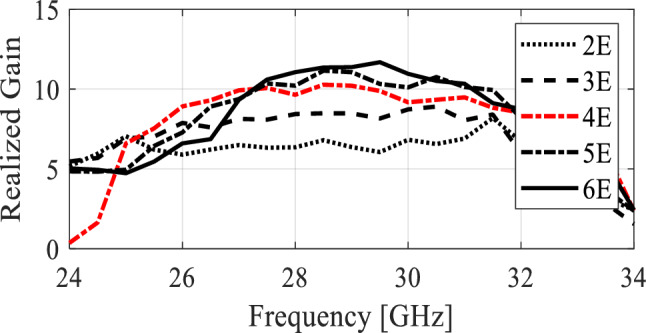
Realized gain of the antenna with different number of elements.

It should be noted that the proposed design offers a versatile balance between gain and physical size. In scenarios with limited space, the antenna can be designed with fewer elements (E) to reduce its footprint, while for applications where circuit size is not a concern, more elements can be added. Figure 7 depicts the achieved gain of the antenna with varying numbers of elements from 2 to 6 elements. Each pair of stub is considered as a single element. The plots display realized gain corresponding to different numbers of elements. For a four-element array, the realized gain surpasses 10 dBic, while a six-element array can achieve close to 12 dBic. Further increasing the number of elements to seven shows trivial gain change due to input signal fading. Therefore, in this paper, four element design (with red color curve) is selected which has relatively high gain and a reasonably compact size. All the geometrical parameters for the four element design are fully optimized at the full-wave level of description.

#### Parametric analysis

**Figure 8:**
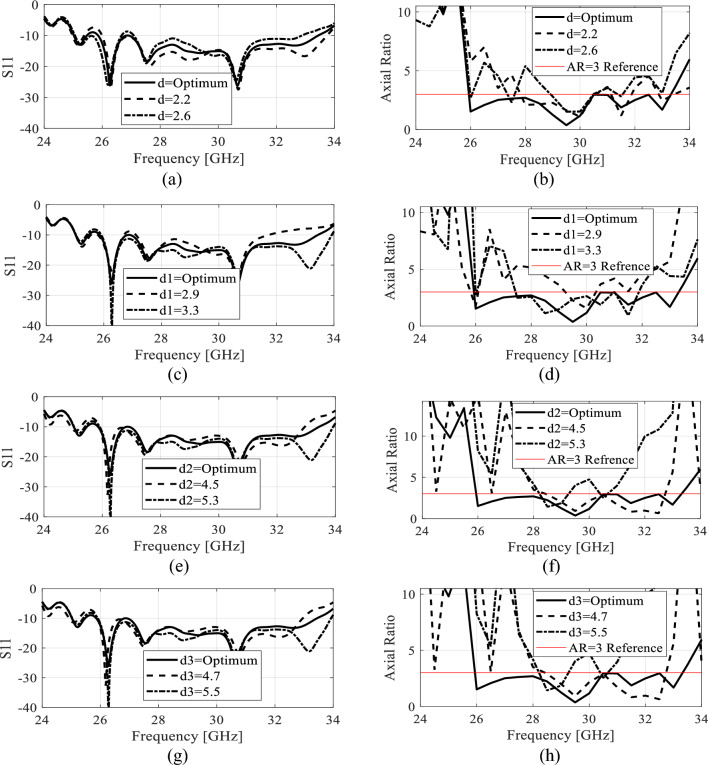

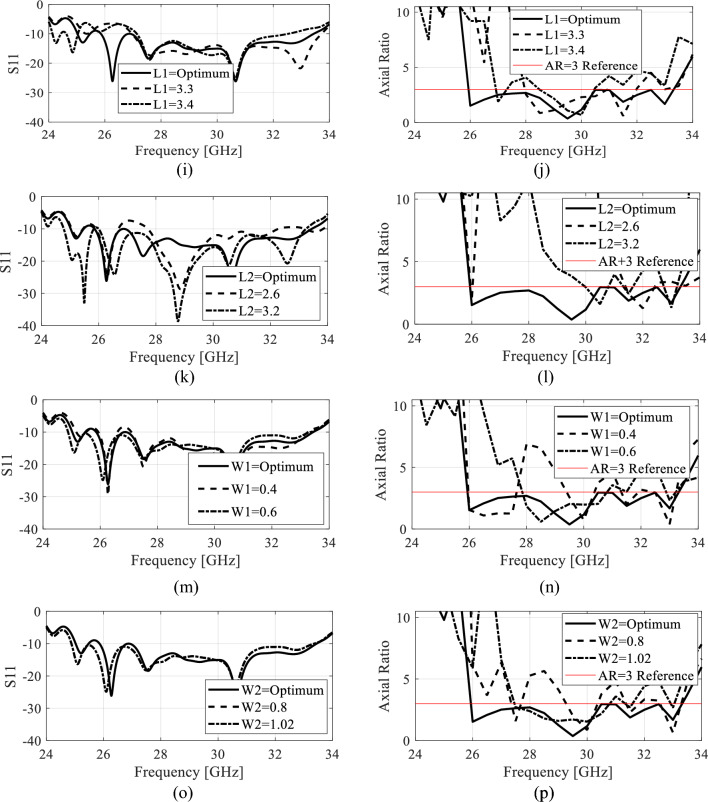
Parametric analysis of different geometrical parameters of the antenna (**a**) S_11_ variation with parameter *d,* (**b**) AR variation with parameter *d*, (**c**) S_11_ variation with parameter *d*_*1*_*,* (**d**) AR variation with parameter *d*_*1*_, (**e**) S_11_ variation with parameter *d*_*2*_*,* (**f**) AR variation with parameter *d*_*2*_, (**g**) S_11_ variation with parameter *d*_*3*_*,* (**h**) AR variation with parameter *d*_*3*_, (**i**) S_11_ variation with parameter *L*_1_*,* (**j**) AR variation with parameter *L*_1_, (**k**) S_11_ variation with parameter *L*_2_*,* (**l**) AR variation with parameter *L*_2,_ (**m**) S_11_ variation with parameter *W*_1_*,* (**n**) AR variation with parameter *W*_1,_ (**o**) S_11_ variation with parameter *W*_2_*,* (**p**) AR variation with parameter *W*_2_.

In this sections, a thorough parametric analysis of the antenna is conducted to gain insights into its key performance metrics and to comprehend its behavior and electrical characteristics. Note that the optimized values of all the parameters are listed in Table 1. The effect of changing the value of the parameter *d* on *S*_11_ and *AR* is shown in Fig. 8a and b. Increasing *d* from it optimum value lowers the impedance matching as well as the *AR* value deviates from its reference range especially in the lower frequencies. Figure 8c and d shows the effect of changing *d*_1_ on *S*_11_ and *AR*. This parameter has almost similar effect on the electrical characteristics of the antenna as the preceding parameter. The results of changing the value of *d*_2_ are illustrated in Fig. 8e and f. As seen this parameter has little effect on *S*_11_ as the antenna still retain the value impedance matching below -10 dB in the whole operating bandwidth, but the AR is adversely affected in both the upper and lower frequencies of the antenna. Extending the discussion further, we look into the effect of *d*_3_ in Fig. 8g and h. As seen the change in *d*_3_ has a trivial effect on *S*_11_ value, but the *AR* at the lower end of the operating frequency is significantly impacted, underscoring the sensitivity of these parameters to changes in the *AR*. Finally, the geometrical parameters of the capacitively coupled stubs are analyzed in Fig. 8i, j, k and l. These parameters has a significant impact on the *S*_11_ as well as the *AR* of the antenna. These two parameters controls the amount of (out-of-phase) field cancellation and the constructive addition of the in-phase vector field components. Therefore, they both have noteworthy effect on both the performance figures of the antenna. The effect of the stubs widths (*W*_1_ and *W*_2_) is also studied in terms of reflection and axial ratio. Figure 8m and n shows that the width *W*_1_ has a minor effect on the impedance bandwidth. Changing this parameters slightly shifts the frequency upward or downward. When the *W*_1_ is decreased from its optimum value, the combined current length along the width of the stubs is reduced which adversely effect the AR at the lower operating band of the antenna. As these two parameters controls the width of the overall horizontal current, a similar effect of both the electrical characteristics is observed with the change in *W*_2_ as indicated in Fig. 8o and p. In the proposed work, all the parameters are optimized at the full-wave level of description to ensure optimum performance of the antenna in terms of impedance bandwidth and AR.

### Experimental validation

**Figure 9:**
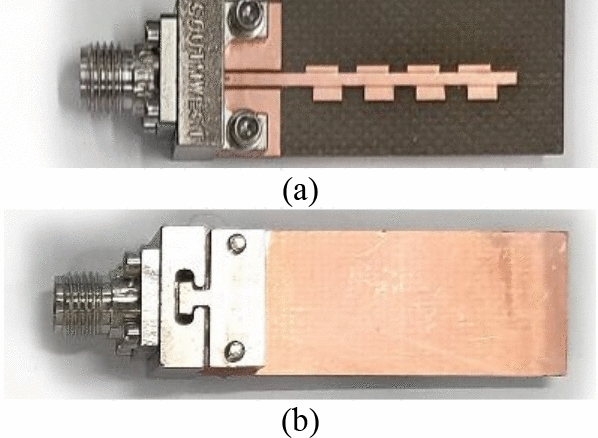
Photographs of the fabricated prototype of the proposed antenna with end-launch Southwest connector: (**a**) front view, (**b**) back view.

**Figure 10:**
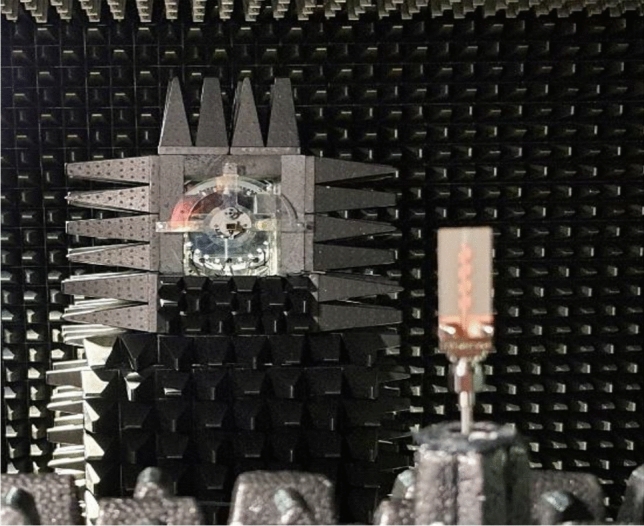
Antenna in the anechoic chamber for experimental validation.

The computational model of the proposed antenna has been numerically characterized at the full-wave level of description using CST Microwave Studio. Following an extensive numerical analysis, a prototype of the antenna has been fabricated and characterized experimentally. Photographs of the physical design are shown in Fig. 9, whereas the experimental setup in the anechoic chamber is shown in Fig. 10. The radiation pattern has been measured using the Pasternack horn antenna PE9850/2F-15 and Anritsu MS2644B 0–40 GHz vector network analyzer. The distance between measurement towers (or between the reference and the measured antennas) is about three meters, whereas the far field distance at 26 GHz is less than 10 cm.

The antenna is fed with a Southwest end-launch connector (1092-03A-6), which is mounted on the antenna for experimental validation. The electrical and field performance figures of the antenna are characterized according to the IEEE antenna measurement standard (IEEE Std149-1979 (R2008)). In the following sub-sections, the proposed antenna's simulated and measured results are discussed in detail.

#### Impedance bandwidth axial ratio characterization

**Figure 11:**
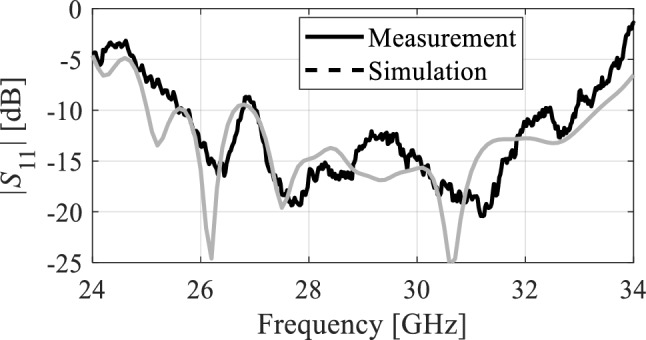
EM-simulated (gray) and measured (black) reflection coefficient.

**Figure 12:**
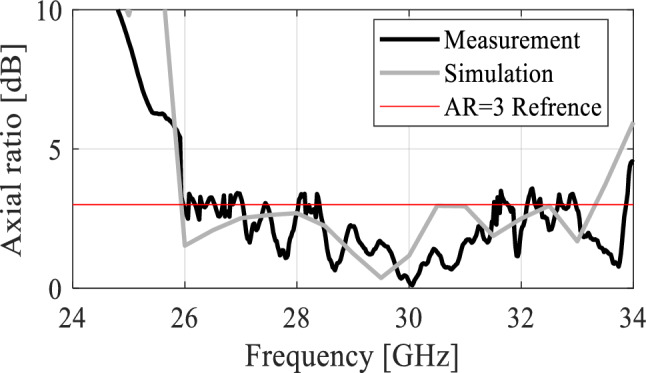
EM-simulated (gray) and measured (black) axial ratio of the proposed planar antenna array.

The impedance bandwidth of the proposed antenna is shown in Fig. 11. The simulation data is depicted with a solid gray line while the measured data is in solid black to clearly differentiate between the two. It can be observed that the simulated reflection coefficient *S*_11_ is less than − 10 dB from 25 to approximately 33 GHz corresponding to 8 GHz impedance bandwidth. Likewise, the measured data closely matches the simulation results. The measured *S*_11_ is below − 10 dB from roughly 25 to 33.5 GHz reflecting the wide bandwidth of the antenna. Moreover, these results clearly indicate good impedance matching (achieved without any additional impedance matching circuit) from the input to the microstrip line radiator. As mentioned in the previous section, the proposed antenna is circularly polarized. The axial ratio of the antenna is numerically and experimentally characterized in the broadside direction and the results are illustrated in Fig. 12. Both the simulated and measured data show that the proposed antenna is circularly polarized for almost 95% of the impedance bandwidth of the antenna. As can be observed, the axial ratio is mostly below 3 dB from 26 to 33.5 GHz with minor discrepancies between EM simulations and measurements.

#### Realized gain and radiation pattern analysis

**Figure 13:**
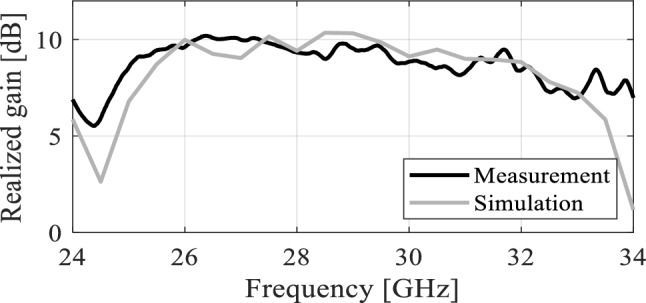
EM-simulated (gray) and measured (black) realized gain (maximum gain in the *zy*-plane).

**Figure 14:**
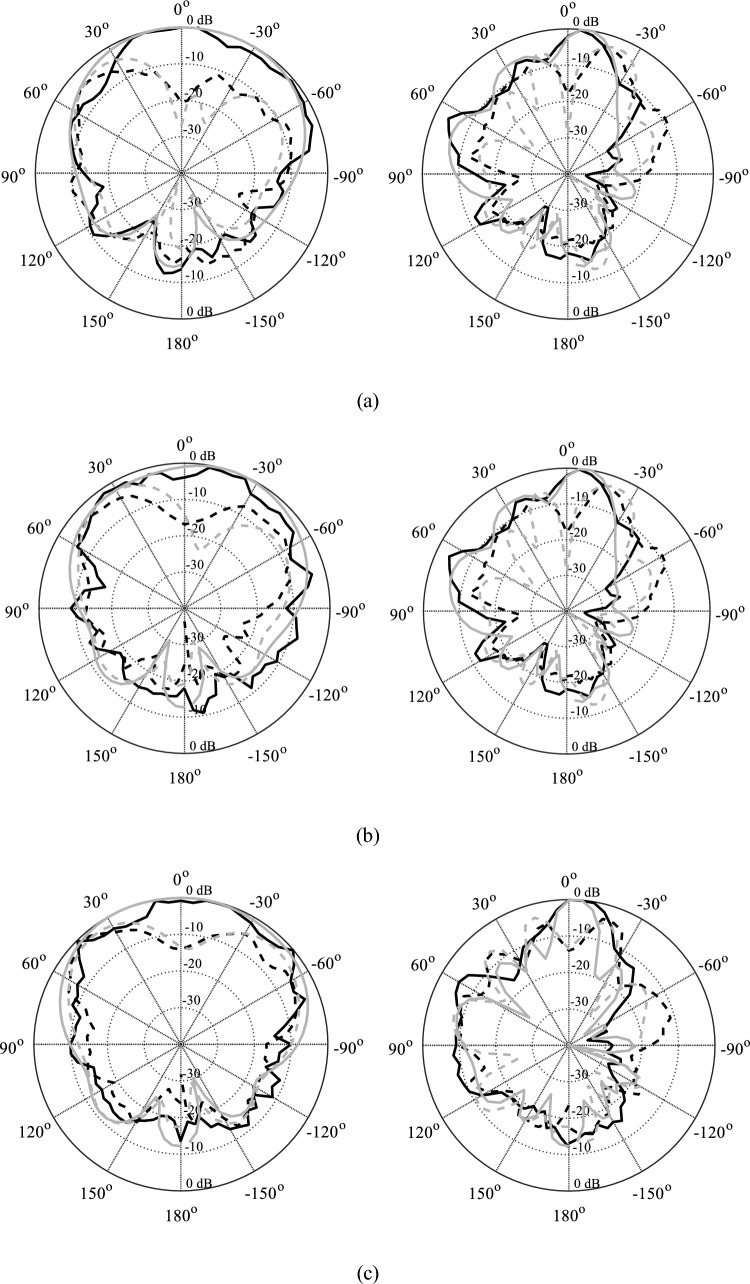
Simulated (gray) and measured (black) *xz*- (left) and *yz*-plane (right) patterns for: (**a**) 27 GHz, (**b**) 29 GHz, (**c**) 31 GHz; LHCP (—), RHCP (-—-).

The realized gain of the proposed antenna is analyzed numerically as well as experimentally in the broadside direction and the results are shown in Fig. 13. The average in-band realized gain is roughly 9.5 dBic. The simulated peak gain is 10.3 dBic, while the measured peak value of the peak gain is roughly 10.1 dBic. Both datasets are in close agreement and show a relatively flat gain with an average variation of 1.3 dB, in the entire operating band of the antenna. The radiation pattern of the antenna is another important performance figure, particularly in the mm-wave band, where propagation losses are relatively high compared to the lower frequency spectrum. The proposed antenna is characterized at several frequencies to ensure far-field stability. Figure 14 illustrates the radiation patterns of the antenna at multiple frequency points taken at 27 GHz, 29 GHz, and 31 GHz. The LHCP and RHCP fields are plotted for both the *xy*- and *yz*-planes. The radiation pattern is mainly pointed in the broadside direction with a minor beam tilt which is due to the size difference, and hence asymmetric current distribution on the capacitively coupled stubs. The LHCP fields are more than 10 dB stronger with respect to RHCP, indicating reliable radiation and high polarization purity of the antenna and acceptable level of in-band cross-pole discrimination^27,28^. Moreover, only a slight beam tilt is observed in the main beam with the change in frequency which ensures a highly stable radiation pattern across the operating bandwidth. The 3 dB AR beamwidth of the antenna is evaluated at different frequencies. The overall in-band AR beamwidth is ± 5° in the broadside direction which is greatly suitable for applications requiring focused coverage and high power efficiency^29,30^. At 30 GHz a broader beamwidth is observed where the AR is very low at the broadside, with a corresponding beamwidth of around ± 10 degrees.

#### Comparison with the state-of-the-art designs

The performance of the proposed antenna has been compared with the recent state-of-the-art designs in Table 2. All major electrical features including the operating frequency, polarization, axial ratio and impedance bandwidth, peak realized gain, and the substrate used are considered in the comparison table. The quantitative analysis of all the performance figures clearly indicates the superiority of the proposed structure over the recently published designs. It is important to note here that several designs in the literature exhibit better performance, cf. ^2,3,5,7,12,13,21,31,32^. Nevertheless, these designs are realized using complex multilayer methods and/or utilizing expensive feeding mechanisms involving active circuit elements. Additionally, the majority of the designs mentioned earlier exhibit a limited bandwidth and operate using linear polarization. Reference^10^ proposes a single-element design with a wider impedance bandwidth, but this design not only has a smaller gain but also utilizes metallic vias, which adds to the complexity of the design. Similarly, references^33^ and^34^ feature complex geometrical configuration that encompasses waveguide excitation feeding, large aperture size, and the employment of multilayer structures. In contrast, the suggested design is implemented on a single substrate, features a simple geometry, and it does not require any active components to generate circularly polarized fields. On the other hand, the proposed design is realized on a single substrate featuring a simple geometry and does not involve any active elements for the excitation of circularly polarized fields. Additionally, a size comparison is done in terms of free space wavelength calculated at the center frequency. For a fair comparison, we evaluate the size of the single antenna, aligning with the reference antenna, which primarily features single antenna designs. It's worth noting that the antenna referenced in^35^ is considerably smaller in size, but its other electrical characteristics are not comparable to those of the proposed antenna.

**Table 2:** Comparison with the state-of-the-art designs.

Refs.	Operating frequency (GHz)	No of elements	% |*S*_11_| BW	CP/LP	Peak gain	Substrate permittivity	Size (L × W) (λ_o_ × λ_o_)
^10^	28	1	46.4	CP	6.5	2.2	3.92 × 1.4
^16^	27	4	21.4	LP	12.8	2.2	2.70 × 0.90
^17^	24	26	3.3	LP	15	3.55	8.97 × 1.87
^20^	76	8	13.4	LP	10.4	3	–
^21^	28	1	16	CP	8.8	3.3	3.8 × 1.25
^22^	24	–	5	CP	8.5	3.3	7.59 × 1.09
^35^	28	1	8	CP	2.2	2.2	0.19 × 0.15
^33^	60	1	17.9	CP	14.2	2.2	30 × 20
^34^	30	16	40.21	CP	20.2	2.2	–
This work	28	4	28	CP	10.3	3.38	2.8 × 0.94

## Conclusion

A single-layer series-fed array antenna operating with circular polarization is presented in this paper for IoT applications. The antenna is based on a modified microstrip line extended from a coplanar waveguide in the *y*-direction. The conventional microstrip lines have symmetric fields with equal magnitude but opposite directions resulting in field cancelation along the length of the line. In the paper, four pairs of stubs are capacitively coupled in the ± *x*-direction to the microstrip line at every half-guided wavelength with a slight offset in the *y*-direction. The addition of the stubs induces amplitude tapering resulting in the alteration of the symmetric fringing electric and magnetic fields. Moreover, the offset in the capacitively coupled stubs enables excitation of circularly polarized fields in the wide frequency spectrum yielding a wideband antenna that operates with circular polarization. The advantages of the proposed design include simple geometry, planar structure (which can be easily integrated), ease of excitation, and low fabrication complexity. Given the achieved performance in terms of electrical characteristics and the mentioned topological advantages, the proposed antenna can be considered a potential candidate for IoT devices and sensors in the mm-wave band.

## Data Availability

All data generated or analyzed during this study is included in this article.
